# Supplementary material for: Promoting routine syphilis screening among men who have sex with men in China: study protocol for a randomised controlled trial of syphilis self-testing and lottery incentive
Source: BMC Infect Dis. 2020 Jun 29;20:455. doi: 10.1186/s12879-020-05188-z (PMC7325146; doi:10.1186/s12879-020-05188-z)
Supplement: Supplementary file 1 — Additional file 1. Results of the pilot study: Table 1. Socio-demographics of the three groups. Table 2. Risk ratios for syphilis, HIV and Other STIs testing uptake, Standard SST and Lottery SST versus control. Table 3. Syphilis testing methods used among men who have sex with men during study period by study groups. [file 12879_2020_5188_MOESM1_ESM.docx]

Table 1: Socio-demographics of the three groups.

| Characteristics | Control group (N=48)  n（%） | Standard SST group (N=49)  n（%） | Lottery SST group  (N=48)  n（%） | *P* |
| --- | --- | --- | --- | --- |
| Age |  |  |  | 0·96^*^ |
| 18-25 | 32 (66.6) | 31 (63.3) | 32 (66.6) |  |
| 26-35 | 14 (29.2) | 16 (32.7) | 13 (27.1) |  |
| 36+ | 2 (4.2) | 2 (4.1) | 3 (6.3) |  |
| Educational attainment |  |  |  | 0.06 |
| Under college | 35 (72.9) | 25 (51.0) | 26 (54.2) |  |
| Bachelor’s degree and above | 13 (27.1) | 24 (49.0) | 22 (45.8) |  |
| Monthly income (CNY) |  |  |  | 0.05 |
| Less than 3000 | 24 (50.0) | 18 (36.7) | 24 (50.0) |  |
| 3001-5000 | 17 (35.4) | 11 (22.4) | 13 (27.1) |  |
| 5001 above | 7 (14.6) | 20 (40.8) | 11 (22.9) |  |
| Marital status |  |  |  | 1.00 |
| Never married | 44 (91.7) | 45 (91.8) | 44 (91.7) |  |
| Ever married/engaged | 4 (8.3) | 4 (8.2) | 4 (8.3) |  |
| Self-reported sexual orientation | | |  | 0.84 |
| Homosexual | 38 (79.2) | 39 (79.6) | 36 (75.0) |  |
| Bisexual/other | 10 (20.8) | 10 (20.4) | 12 (25.0) |  |
| Ever tested for syphilis |  |  |  | 0.35 |
| Yes | 10 (20.8) | 13 (26.5) | 7 (14.6) |  |
| No | 38 (79.2) | 36 (73.5) | 41 (85.4) |  |
| Ever received an HIV test |  |  |  | 0.29 |
| Yes | 30 (62.5) | 35 (71.4) | 27 (56.3) |  |
| No | 18 (37.5) | 14 (28.6) | 21 (43.7) |  |
| Ever used an HIV self-test |  |  |  | 0.21 |
| Yes | 25 (52.1) | 28 (57.1) | 19 (39.6) |  |
| No | 23 (47.9) | 21 (42.9) | 29 (60.4) |  |
| Disclosure of sexual orientation to Health provider |  |  |  | 0.53 |
| Yes | 12 (25.0) | 14 (28.6) | 17 (35.4) |  |
| No | 36 (75.0) | 35 (71.4) | 31 (64.6) |  |
| Disclosure of sexual orientation to family or friends |  |  |  | 0.06 |
| Yes | 20 (41.7) | 12 (24.5) | 10 (20.8) |  |
| No | 28 (58.3) | 37 (75.5) | 38 (79.2) |  |
| Number of male sex partners in the past three months |  |  |  | 0.69^*^ |
| 0 | 3 (6.3) | 4 (8.2) | 6 (12.5) |  |
| 1 | 13 (27.1) | 14 (28.6) | 10 (20.8) |  |
| 2-3 | 23 (47.9) | 17 (34.7) | 22 (45.8) |  |
| 4~ | 9 (18.8) | 14 (28.6) | 10 (20.8) |  |
| Sex role |  |  |  | 0.41 |
| Mostly insertive | 11 (22.9) | 16 (32.7) | 18 (37.5) |  |
| Mostly receptive | 18 (37.5) | 19 (38.7) | 19 (39.6) |  |
| Both | 19 (39.6) | 14 (28.6) | 11 (22.9) |  |
| Female sex partner in the past three months |  |  |  | 0.91^*^ |
| Yes | 5 (10.4) | 4 (8.2) | 4 (8.3) |  |
| No | 43 (89.6) | 45 (91.8) | 44 (91.7) |  |
| Had used substances before or during sex in the past three months |  |  |  | 0.10 |
| Yes | 29 (60.4) | 19 (38.8) | 24 (50.0) |  |
| No | 19 (39.6) | 30 (61.2) | 24 (50.0) |  |
| Had group sex in the past three months |  |  |  | 0.80 |
| Yes | 5 (10.4) | 7 (14.3) | 7 (14.6) |  |
| No | 43 (89.6) | 42 (85.7) | 41 (85.4) |  |
| Condomless anal intercourse with regular partner in 3 months prior to the study | | | | 0.95 |
| Yes | 26 (54.2) | 26 (53.1) | 27 (56.2) |  |
| No | 22 (45.8) | 23 (46.9) | 21 (43.8) |  |
| Condomless anal intercourse with casual partner in 3 months prior to the study | | | | 0.66 |
| Yes | 22 (45.8) | 22 (44.9) | 18 (37.5) |  |
| No | 26 (54.2) | 27 (55.1) | 30 (62.5) |  |

Table 2 Risk ratios for syphilis, HIV and Other STIs testing uptake, Standard SST and Lottery SST versus control.

|  | Tested rate | OR (95% CI) | AOR (95%CI)^a^ |
| --- | --- | --- | --- |
| Tested for syphilis in past one month  Control  Standard SST  Lottery SST | 16 (36.4)  32 (74.4)  28 (70.0) | 1.00  5.09 (2.03 to 12.78) ^*^  4.08 (1.64 to 1018) ^*^ | 1.00  6.40 (2.32 to 17.67) ^*^  5.71 (2.06 to 15.82) ^*^ |
| Tested for HIV in past one month  Control  Standard SST  Lottery SST | 16 (36.4)  31 (72.1)  26 (65.0) | 1.00  4.52 (1.83 to 11.19) ^*^  3.25 (1.33 to 7.95) ^*^ | 1.00  5.33 (2.02 to 14.07) ^*^  3.98 (1.52 to 10.45) ^*^ |
| Tested for other STIs in past one month  Control  Standard SST  Lottery SST | 7 (15.9)  6 (14.0)  4 (10.0) | 1.00  0.86 (0.26 to 2.80)  0.59 (0.16 to 2.18) | 1.00  0.81 (0.23 to 2.79)  0.58 (0.15 to 2.31) |

Data given as number (percent).

^a^ Adjusted for educational attainment, monthly income and disclosure of sexual orientation to family or friends.

^*^ *P*<0·01.

^*^ Other STIs included CT, NT, HPV, and HSV.

Table 3 Syphilis testing methods used among men who have sex with men during study period by study groups.

|  | Control group (N=44) | Standard SST group (N=43) | Lottery SST group  (N=40) | Total  (N=127) |
| --- | --- | --- | --- | --- |
| Self-test | 3 (6.8) | 27 (62.8) | 22 (55.0) | 52 (40.9) |
| Facility-based Test | 12 (27.2) | 1 (2.3) | 0 (0) | 13 (10.2) |
| Both | 1 (2.3) | 4 (9.3) | 6 (15.0) | 11 (8.7) |

Data given as number (percent).
